# Visualizing the Tumor Microenvironment: Molecular Imaging Probes Target Extracellular Matrix, Vascular Networks, and Immunosuppressive Cells

**DOI:** 10.3390/ph17121663

**Published:** 2024-12-10

**Authors:** Hui-Wen Chan, Deng-Yu Kuo, Pei-Wei Shueng, Hui-Yen Chuang

**Affiliations:** 1 Department of Biomedical Imaging and Radiological Sciences, National Yang Ming Chiao Tung University, No. 155, Sec. 2, Li-Nong St., Beitou Dist., Taipei City 112, Taiwan; viator70757@gmail.com; 2 Division of Radiation Oncology, Department of Radiology, Far Eastern Memorial Hospital, New Taipei City 220, Taiwan; 3 Faculty of Medicine, School of Medicine, National Yang Ming Chiao Tung University, Taipei City 112, Taiwan

**Keywords:** tumor microenvironment, molecular imaging, PET, MRI, theranostics, imaging probes

## Abstract

The tumor microenvironment (TME) is a critical factor in cancer progression, driving tumor growth, immune evasion, therapeutic resistance, and metastasis. Understanding the dynamic interactions within the TME is essential for advancing cancer management. Molecular imaging provides a non-invasive, real-time, and longitudinal approach to studying the TME, with techniques such as positron emission tomography (PET), magnetic resonance imaging (MRI), and fluorescence imaging offering complementary strengths, including high sensitivity, spatial resolution, and intraoperative precision. Recent advances in imaging probe development have enhanced the ability to target and monitor specific components of the TME, facilitating early cancer diagnosis, therapeutic monitoring, and deeper insights into tumor biology. By integrating these innovations, molecular imaging offers transformative potential for precision oncology, improving diagnostic accuracy and treatment outcomes through a comprehensive assessment of TME dynamics.

## 1. Introduction

**Figure 1:**
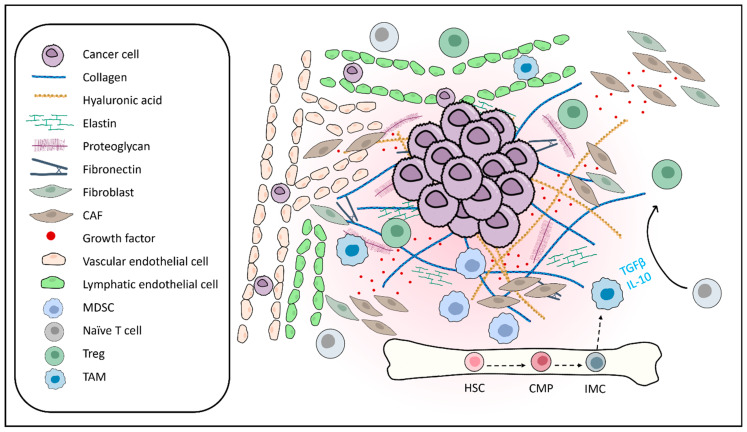
Composition of the Tumor Microenvironment. The tumor microenvironment (TME) comprises cancer cells, the extracellular matrix (ECM), stromal cells, and diverse immune cell populations, all of which collectively influence tumor progression. The ECM provides structural support and regulates cell behavior, with abnormal remodeling promoting stiffness, invasion, and metastasis. Stromal cells, including cancer-associated fibroblasts (CAFs), modulate ECM structure, secrete growth factors, and contribute to immunosuppression. Immune cells such as myeloid-derived suppressor cells (MDSCs), tumor-associated macrophages (TAMs), and regulatory T cells (Tregs) foster immune suppression and support tumor growth. Together, these components interact dynamically, reshaping the mechanical structure, altering cytokine and chemokine profiles, and facilitating immune evasion to drive tumor progression and therapeutic resistance.

Cancer remains the leading cause of death worldwide, claiming millions of lives annually. Mounting evidence reveals that tumor progression is driven not solely by cancer cell proliferation but also by intricate interactions within the tumor microenvironment (TME) [1]. The concept of the TME was first proposed by Stephen Paget in his seminal “seed and soil” theory in 1889 [2,3], suggesting that the success of metastasis relies on both the inherent characteristics of cancer cells (seed) and the supportive microenvironment (soil). Various non-cancerous components can be found in the TME [4] (Figure 1), including cancer-associated fibroblasts (CAFs), immune cells, endothelial cells, and extracellular matrix elements (ECM), which collectively contribute to cancer survival, proliferation, immune evasion, therapeutic resistance, and metastasis [5,6].

Targeting specific TME components presents an ideal strategy for improving cancer treatment and diagnosis. The TME is highly dynamic and continues to evolve throughout cancer progression. In other words, technologies that can identify the early changes in the TME, such as liquid biopsy [7,8] and molecular imaging [9,10], are invaluable tools for diagnosing cancer and monitoring these changes throughout different stages.

Molecular imaging techniques, including positron emission tomography (PET), single-photon emission computed tomography (SPECT), magnetic resonance imaging (MRI), computed tomography (CT), ultrasound, and fluorescence imaging, are widely used in clinical and preclinical cancer research [10]. Each imaging modality offers unique strengths tailored to specific requirements, yet they also present inherent challenges that must be addressed when evaluating their utility for tumor imaging.

PET and SPECT are highly sensitive techniques that detect biochemical and functional changes within the TME. PET imaging excels in quantitative molecular imaging, providing high sensitivity and temporal resolution for tracking specific molecules, including metabolic activity, receptor dynamics, and enzyme activity [11,12]. Similarly, SPECT enables the imaging of molecules labeled with longer-lived isotopes. Both modalities lack anatomical information, often requiring integration with CT or MRI for precise localization. Additionally, using radiotracers with short half-lives can present challenges, particularly for facilities without an on-site cyclotron.

MRI provides exceptional soft tissue contrast and high spatial resolution, making it particularly effective for delineating tumor margins and TME [13]. By adjusting sequencing parameters, MRI can generate images with different signal weightings, such as T1-weighted (T1WI) and T2-weighted (T2WI) imaging, allowing for the monitoring of anatomical and molecular information regarding lesions. Furthermore, MRI does not involve ionizing radiation, making it safer for repeated use in longitudinal studies and treatment monitoring. However, significant drawbacks include its relatively high cost, longer acquisition times, and limited availability. Although MRI can provide some molecular information, its sensitivity is lower than that of PET or SPECT. New contrast agents and pulse sequences need to be developed to enhance its capabilities.

CT is a widely available modality known for its exceptional anatomical detail and fast acquisition times, making it particularly valuable in emergencies and dynamic imaging settings. Its clinical applications include assessing perfusion in ischemic stroke, evaluating coronary calcification, and detecting lung nodules for early cancer diagnosis. However, CT relies on ionizing radiation, which limits the rationality of using it for repeated imaging.

Ultrasound (US) is an accessible and cost-effective imaging method recognized for its ability to provide real-time images without the use of ionizing radiation. It helps guide interventional procedures and monitor fetal development. However, its effectiveness is highly operator-dependent, and its limited penetration depth and resolution reduces its utility for imaging deep-seated tumors or providing detailed insights into the TME. Additionally, the current US contrast agents mainly enhance signal intensity and cannot help to provide molecular details unless labeled with specific ligands.

Fluorescence imaging has gained attention for its applications in intraoperative guidance and preclinical studies [14,15]. It offers non-radiative, real-time visualization of specific molecular targets, significantly enhancing surgical precision. However, its limited tissue penetration and susceptibility to autofluorescence restrict its use to superficial or near-surface applications.

Each imaging technique offers specific advantages tailored to distinct clinical needs (listed in Table 1), and their combination enhances the overall effectiveness of tumor imaging by addressing individual limitations. These technologies are invaluable for early disease detection, disease progression monitoring, therapeutic response assessment, and deepening our understanding of TME interactions at cellular and molecular levels.

The potential clinical impact of these imaging technologies goes beyond diagnostics. Their ability to provide detailed, patient-specific information about TME composition and dynamics plays a crucial role in the era of personalized medicine. By identifying specific molecular and cellular characteristics of the tumors, these techniques facilitate tailored treatment selection, such as choosing patients eligible for immunotherapies, anti-angiogenic agents, or tumor stromal-targeting drugs. Additionally, molecular imaging offers real-time monitoring of therapeutic responses, allowing for adjustments to treatment plans at early time points and ultimately improving patient outcomes.

Specialized imaging probes further enhance the utility of these modalities. Probes targeting key TME components, such as immune cell markers or ECM elements, provide deeper insights into tumor biology and therapeutic resistance. Their application enables precise disease characterization, promoting a shift toward predictive and adaptive cancer management.

In summary, integrating advanced molecular imaging technologies into clinical practice has transformative potential for cancer care. By bridging the gap between tumor biology and treatment, these tools offer a comprehensive approach to diagnosing, monitoring, and personalizing therapy, ultimately leading to improved patient outcomes in oncology. This review summarizes recent advancements in designing innovative probes that target the TME for cancer diagnostics and treatment.

## 2. Imaging the Extracellular Matrix

The extracellular matrix (ECM) is a complex, non-cellular network composed of collagens, proteoglycans, elastin, fibronectin, oxidases, and proteases [16]. The ECM offers several essential physical functions [17], including structural support, maintaining tissue integrity, water retention, mechanical resilience, and protection barriers. Beyond these functions, ECM remodeling plays a critical role in tissue repair [18], regulating cell behavior [19], tissue homeostasis [20], and signal transduction activation [21].

ECM remodeling is a multi-step process involving (1) ECM deposition and modification, which adjusts the biochemical and mechanical properties of ECM by altering its composition; (2) ECM degradation, mediated by proteolytic enzymes such as matrix metalloproteinases (MMPs), leading to the release of ECM fragments and ECM-bound cytokines and ultimately facilitating cell migration; (3) mechanics-mediated ECM remodeling, where integrin-ECM interactions induce deformation and alignment of ECM fibers, thereby modulating the overall ECM structure [16,22].

Cancer cells strategically manipulate ECM remodeling to create a more supportive TME at various stages of tumor progression [20]. This remodeling involves several processes: the differentiation of cancer-associated fibroblasts (CAFs) through stromal cell activation [23], crosslinking CAFs by ECM-modifying enzymes to increase matrix stiffness [24], and the alignment of collagen fibers to establish physical barriers that prevent cytotoxic immune cell infiltration [25,26]. Additionally, ECM-bound cytokines released during ECM remodeling further stimulate angiogenesis and support tumor outgrowth [27]. Both basement membrane degradation and integrin-mediated mechanical forces are critical drivers of cancer invasion and distal metastasis [27,28].

Beyond establishing the tumor niche, ECM remodeling also contributes to treatment resistance [29,30]. Developing ECM-targeting imaging probes can significantly improve cancer diagnosis, guide treatment selection, and predict metastatic potential. This section focuses on molecular imaging probes targeting specific ECM components, including collagen, elastin, and fibronectin.

### 2.1. Collagen

Collagen is the most abundant ECM component in tissues [31], and its abnormal accumulation is associated with increased cancer risk [32]. As a result, collagen has emerged as a potential prognostic marker for cancer patients [33], and collagen-targeted therapies may help slow tumor progression and prevent metastasis.

Desogere et al. developed a series of collagen-binding probes (CBPs) for imaging pulmonary fibrosis [34,35]. Among all the CBPs, [^68^Ga]Ga-CBP8 was later used to visualize collagen deposition in pancreatic ductal adenocarcinoma (PDAC) patients undergoing FOLFIRINOX treatment using PET/MRI. In PDAC-bearing mice, PET revealed a strong positive correlation between tumor collagen deposition and [^68^Ga]Ga-CBP8 uptake, mirroring the results detected in a PDAC patient with FOLFIRINOX-induced collagen accumulation. These results suggest the potential of [^68^Ga]Ga-CBP8 for monitoring chemotherapy response non-invasively [36].

The same group also developed EP-3533, a gadolinium (Gd)-labeled collagen-detecting probe for MRI [37]. Enhanced MRI signals indicating collagen deposition in LNCaP prostate tumors were validated through Picrosirius Red staining at the histological level [38]. Additionally, Guo et al. introduced a collagen-responsive indocyanine green (ICG) probe, the collagen-adhesive probe (CA-P), for detecting collagen distribution in bladder cancer via a sol–gel transformation triggered by the interactions between CA-P and collagen [39]. The probe utilizes aldehyde groups on a hydrogel to form a stable gel with amine-rich regions in collagen through a Schiff base reaction [40]. CA-P effectively helped delineate tumor boundaries and increase surgical precision [34]. These imaging probes demonstrate the potential of targeting collagen for cancer diagnosis, treatment monitoring, and image-guided surgery, underscoring its value in advancing precision oncology.

### 2.2. Elastin

Elastin provides essential elasticity and resilience to various tissues such as skin, lungs, and blood vessels [41]. Recent studies reveal that elevated elastin and collagen fiber accumulation correlates with increased incidence and poorer prognosis in cancers such as hepatocellular carcinoma and gastric cancer [42,43].

Keller et al. developed a Gd-labeled elastin-specific molecular agent (Gd-ESMA), a low-molecular-weight MRI contrast agent, to visualize the TME in VX2 HCC-bearing rabbits. Compared to a conventional Gd-based MRI contrast agent gadobutrol, elastin-specific ESMA resulted in significantly stronger signal enhancements in the tumor center, tumor margins, and peritumoral regions [44]. Gd-ESMA has also been applied to monitor the dynamic changes in elastin deposition in VX2 tumor-bearing rabbits following radiofrequency ablation, offering insight into TME remodeling post-treatment [45]. Additionally, in PC3 prostate cancer-bearing mice, higher Gd-ESMA signals were detected with smaller tumors, compared to the ones with larger tumors, indicating more elastin deposition within tumors and possibly increased metastatic potential [46].

A radiolabeled elastin-targeting probe was also developed, using elastin-like polypeptides labeled with iodine-131. This theranostic agent leverages the γ-ray and β-particles emitted by iodine-131, combining SPECT imaging for elastin-specific visualization with ^131^I-mediated brachytherapy that significantly inhibits tumor growth in VX2 tumor-bearing rabbits [47].

Since elastin deposition influences TME remodeling and correlates to distal metastasis, elastin-targeting probes offer a promising cancer imaging and treatment approach. These probes enhance our understanding of tumor biology, facilitate early cancer detection, enable non-invasive monitoring of tumor progression, and have potential applications in theranostics for precision oncology through targeting elastin.

### 2.3. Fibronectin

Fibronectin is a glycoprotein vital for regulating cell adhesion, wound healing, and tissue repair and providing a scaffold for cell attachment and migration [48]. The above physiological processes are crucial for cell growth, differentiation, and survival. However, abnormal fibronectin production and reorganization are frequently observed in cancer, where they are closely linked to tumor progression, invasion, and metastasis [49,50]. Changes in fibronectin play a critical role in establishing the “pre-metastatic niche”, a microenvironment that enhances colonization and survival of circulating tumor cells, thereby promoting distant metastasis [51]. Fibronectin alterations also contribute to angiogenesis, facilitating new blood vessel formation that nourishes cancer cells and sustains their growth [52]. Accordingly, fibrin–fibronectin complexes have emerged as important imaging biomarkers [53], enabling precise cancer detection with specific imaging probes.

Zhou et al. developed a tumor-homing pentapeptide, Cys-Arg-Glu-Lys-Ala (CREKA), which targets fibrin–fibronectin complexes in the TME. By labeling it with gadolinium, they synthesized CREKA-Tris(Gd-DOTA)_3_ for MRI. Compared to the conventional MRI agent Prohance™, CREKA-Tris(Gd-DOTA)_3_ doubled the contrast-to-noise ratio and maintained tumor signal visibility for at least 30 min [54]. The detection limit of the probe was further tested in a 4T1 breast cancer mouse model of spontaneous metastasis. CREKA-Tris(Gd-DOTA)_3_; successfully identified bone micrometastases smaller than 0.5 mm in diameter, with a sensitivity of 79% for tumors as small as 0.125 mm^3^ [55].

Yang et al. modified the CREKA probe by conjugating it with a near-infrared fluorophore (Cy7), creating a dual-contrast agent, CREKA-Cy7(Gd-DOTA)_3_. This modified probe improved the visualization of HGC-27 gastric cancers in vivo, providing enhanced sensitivity and reduced imaging time [56]. Additional modifications to fibronectin-targeting probes have further increased tumor specificity and precision. For example, Cheng et al. developed an MMP-activatable probe, CREKA-GK8-QC, which combined fibronectin specificity with MMP-9 responsiveness. This probe demonstrated superior tumor retention and specificity compared to its non-fibronectin-targeting counterparts, enabling better identification of microscopic cancer lesions in 4T1 tumor-bearing models [53].

Fibronectin-targeting probes have also been engineered to focus on the extra domain B (ED-B) fibronectin variant, which is selectively expressed during angiogenesis and tumorigenesis [57,58]. The ED-B variant accumulates around neovascular structures in aggressive tumors and remodeling tissues, making it a conserved and highly specific target. Han et al. synthesized ZD2, an ED-B-binding peptide, for near-infrared fluorescence (NIRF) imaging [59]. To overcome the limited tissue penetration of NIRF imaging, Ye et al. radiolabeled ZD2 with technetium-99m, creating [^99m^Tc]Tc-HYNIC-ZD2 for SPECT, thereby enhancing its clinical applicability [60].

In addition to peptides, recombinant antibody fragments targeting ED-B have been demonstrated to have promising applications in clinical trials. Radretumab (L19SIP), an ED-B-specific antibody fragment, has been successfully used for both imaging and radioimmunotherapy in patients with lung, colorectal, or brain cancer. Using [^123^I]I-L19(scFv)_2_/SPECT imaging, liver metastases from colorectal cancer and small-cell lung carcinoma with mammary involvement were effectively visualized [61]. Furthermore, ^131^I-L19SIP has been applied as a radioimmunotherapy agent, while [^124^I]I-L19SIP has been utilized for high-resolution PET imaging and accurate dosimetry, particularly for red bone marrow [62]. Tijink et al. reported that [^131^I]I-L19SIP exhibited significantly lower accumulation in the liver, spleen, and kidneys compared to ^177^Lu-labeled L19SIP [58]. Furthermore, [^131^I]I-L19SIP demonstrated superior stability, whereas [^79^Br]Br-L19SIP faced challenges due to debromination, compromising its radiochemical purity. This instability increases the risk of off-target activity accumulation in non-target organs during in vivo applications [63,64].

Fibronectin-targeting probes, including CREKA-based and ED-B-specific agents, hold significant potential in advancing cancer diagnostics and treatment. These imaging probes have demonstrated exceptional precision in tumor detection, intraoperative guidance, and theranostic applications, underscoring their transformative role in enhancing cancer management.

## 3. Molecular Imaging of Stromal Cells

In addition to the ECM and fibronectin, the TME encompasses various stromal cell types recruited from neighboring non-cancerous tissues or derived through the transformation of cancer cells themselves [65]. These stromal cells secrete diverse signaling molecules and exosomes, which play crucial roles in triggering angiogenesis and modulating immune responses—key processes in tumor initiation, progression, and drug resistance [66]. As highlighted, stromal cells, including cancer-associated fibroblasts (CAFs), tumor-endothelial cells, and immune cells [67,68], actively remodel the ECM, and influence critical aspects of tumor biology such as metabolism, growth, metastasis, and immune evasion. Their continuous and dynamic interaction with the ECM not only sustains tumor progression but also enhances therapeutic resistance. Given their vital role in tumorigenesis, tumor-associated stromal cells have emerged as promising therapeutic targets, offering novel avenues to disrupt tumor development and progression while potentially overcoming resistance mechanisms.

### 3.1. Cancer-Associated Fibroblasts

Cancer-associated fibroblasts (CAFs) are the most abundant stromal cells within the TME, typically activated from resident fibroblasts in response to stimuli such as TGF-β [69], platelet-derived growth factor (PDGF) [70], and fibroblast growth factor-2 (FGF-2) [71,72]. Unlike normal fibroblasts, CAFs exhibit dysregulated signaling pathways and altered protein expression profiles [73], enabling them to reshape the TME architecture, hinder drug diffusion, and thereby reduce treatment efficacy [74]. Activated CAFs are defined by the upregulation of specific markers, including α-SMA [75], fibroblast-specific protein 1 (FSP1) [76], and fibroblast activation protein (FAP) [77,78]. These markers not only distinguish CAFs from normal fibroblasts but also highlight their role in driving tumor progression and resistance to therapy, making them promising targets for cancer diagnosis and treatment.

CAFs overexpressing FAP are frequently found within the tumor stroma across various cancer types [77,78]. In contrast, FAP expression is minimal in normal tissues, making it an ideal target for cancer diagnosis and therapy. Due to its enzymatic activity, FAP can cleave specific peptides, enabling the design of innovative imaging agents.

Wu et al. developed a FAP-responsive probe featuring a valine–proline dipeptide conjugated with a cyanine-derived NIR dye, exhibiting excellent FAP-targeting capability. This probe effectively captures FAP-expressing CAFs in LX-2 hepatic stellate cells—the primary source of CAFs in the liver—as well as in HCC-bearing mice. Upon FAP-mediated cleavage, the imaging probe is activated and emits strong NIR signals, demonstrating its potential for both in vitro and in vivo FAP imaging. Notably, FAP-expressing CAFs contribute to immune evasion by promoting reactive oxygen species (ROS) generation and upregulating PD-L1 expression, further underscoring their role in tumor progression and therapeutic resistance [79].

Due to the preferential expression of FAP in CAFs and their scarcity in normal tissues, radiolabeled FAP inhibitors (FAPIs) have emerged as valuable tools for cancer theranostics, particularly in tumors characterized by extensive fibrotic stroma. Several ^18^F- and ^68^Ga-labeled FAPI compounds (e.g., FAPI-04, FAPI-46, and FAPI-74) have shown promising diagnostic potential across multiple cancer types when imaging with PET scanners. In a study involving 62 patients with pancreatic adenocarcinoma, which is characterized by significant desmoplastic responses and associated hypometabolism, [^18^F]FAPI-04 demonstrated superior capabilities in detecting the primary tumor as well as identifying liver, peritoneal, and lymphatic metastases, compared to [^18^F]FDG [80]. Additionally, in a case involving a 52-year-old woman with advanced pancreatic adenocarcinoma, [^68^Ga]Ga-FAPI-46 exhibited a lower background signal, a stronger signal in small lesions, and a higher standard uptake value (SUV) compared to [^18^F]FDG [81].

In lung cancer, compared to [^18^F]FDG, [^18^F]FAPI showed a lower tumor-to-background signal in primary lesions, but high signals in lymphatic and bone metastasis [82]. These FAP-targeting probes allow better visualization of tumor boundaries, more accurate assessment of metastatic spread, and potential therapeutic response monitoring. To enhance tumor retention and tumor-to-tissue ratios, a ^64^Cu-labeled PET imaging probe containing four FAPI-46 motifs attached to diethylene glycol (mini-PEG) spacers was developed. Furthermore, [^177^Lu]Lu-4P(FAPI)_4_ showed enhanced tumor suppression compared to [^177^Lu]-FAPI-46 [83]. The glycopeptide-modified version of FAPI-42, [^18^F]FAPT, exhibited reduced hepatobiliary uptake and twice the tumor accumulation compared to FAPI-42 in A549-FAP tumor-bearing mice [84].

The ^18^F- and ^68^Ga-labeled FAPI probes have been used in a clinic setting, as they can be taken up against multiple cancer types [85,86]. The [^68^Ga]Ga-FAPI probes exhibit remarkable specificity in identifying liver nodules [87] and PDAC [88], which can be difficult to detect using alternative imaging techniques.

Recently, accumulating evidence has shown that [^68^Ga]Ga-FAPI may outperform [^18^F]FDG in revealing lung cancer [89], pancreatic cancer [90], colorectal cancer [91], and nasopharyngeal carcinoma [92]. Furthermore, the high sensitivity and accuracy of [^68^Ga]Ga-FAPI imaging are advantageous for staging and planning radiotherapy [93,94]. An in-depth review of ^18^F- and ^68^Ga-based FAPI PET/CT imaging is also available [95,96].

Another series of FAP-imaging probes have been developed, including PNT6555, PNT6952, and PNT6522, using the amino-terminally blocked D-Ala-boroPro as the targeting moiety. These probes were further radiolabeled with gallium-68, lutetium-177, and terbium-161, and their theranostic potential was verified in HEK-mFAP tumor-bearing mice. Notably, PNT6555, when radiolabeled with actinium-225, demonstrated significant tumor growth inhibition, highlighting the potential of FAP-targeted alpha therapy [97].

Bentivoglio et al. developed a [^99m^Tc]Tc-FGF-2 probe that targets FGFR-2, which is overexpressed on CAFs and TAMs, and tested its efficacy in reticulum cell sarcoma-bearing mice J774A [98]. Additionally, Dai et al. designed a CAF-mimetic aggregation-induced emission (AIE) probe with a CAF-derived cell membrane coating to enhance targeting specificity. The fibronectin present on the CAF membrane enables specific binding to integrin α5 (ITGA5), which is overexpressed in cancer cells. This AIE imaging probe was applied in ovarian cancer surgery, significantly improving lesion detection compared to visual inspection alone, potentially facilitating the optical-guided surgery and increasing precision [99].

CAF-targeted imaging probes, particularly those directed at FAP, have shown remarkable potential in advancing cancer diagnosis and therapy. Radiolabeled FAPIs and FGF-2, along with innovative probes such as CAF-mimetic AIE probes, enable precise visualization of CAFs within the TME. These technologies significantly improve diagnostic accuracy, facilitate response monitoring, and enhance surgical precision by delineating tumor margins more effectively. Given the critical roles of CAFs in tumor progression, immune evasion, and treatment resistance, these advancements highlight the promise of CAF-targeted strategies in revolutionizing cancer imaging and therapeutic interventions.

### 3.2. Vascular and Lymphoid Networks

Blood and lymphatic vessels, formed by endothelial cells (ECs) [100], are essential for transporting blood, facilitating oxygen exchange, and recycling lymphatic fluid. In the TME, these vascular and lymphatic structures are repurposed to support rapid tumor growth and recruit immunosuppressive cells from surrounding tissues and circulation [27,101]. While these structures supply nutrients and oxygen to sustain tumor progression, they are often chaotic and dysfunctional compared to normal vessels, contributing to treatment resistance [102]. Tumor neovasculature is characterized by leaky vessel walls resulting from disrupted cell junctions and a nearly or entirely absent basement membrane. These structural abnormalities reduce the energy required for cancer cells to traverse the vessels, thereby facilitating migration and metastasis [103]. Such aberrant vascular and lymphatic changes highlight their critical roles in cancer progression, which will be discussed in detail below.

#### 3.2.1. Vascular Vessels

To meet the heightened demand for oxygen and nutrients driven by rapid tumor growth, cancer establishes complex vascular networks through two primary mechanisms: vasculogenesis and angiogenesis [104].

Vasculogenesis involves the formation of new vessels from bone marrow-derived endothelial progenitor cells (EPCs) [105,106], which express specific markers such as CD34, VEGFR1/2, CD133, Tie-2, Nanog, and Oct-4 [27,107]. EPCs are recruited to hypoxic tumor regions in response to growth factors, cytokines, and hypoxia-induced signals. Within the TME, elevated HIF-1α drives VEGF expression, which attracts VEGFR2^+^ EPCs to tumor sites and promotes vasculogenesis [108]. Additionally, chemotaxis pathways, including VEGF/VEGFR, CCR2/CCL2 [109], and CCR5/CCL5, are also prominent for recruiting EPCs to the TME.

In contrast, angiogenesis involves the sprouting of new vessels from pre-existing capillaries [110], primarily driven by the VEGF/VEGFR pathway. VEGF generated by cancer cells binds to VEGFR expressed on nearby ECs, activating enzymes such as elastase, MMPs, and cathepsin G. These proteases mediate ECM remodeling, facilitating EC migration, sprouting, and proliferation, ultimately leading to the formation of new blood vessels. Intriguingly, sometimes aggressive cancer cells can also form vessel-like structures, referred to as vascular mimicry [111], which are not derived from ECs but still provide an alternative nutrient supply by mimicking blood vessels.

Tumor vasculature is usually irregular, dysfunctional, and highly permeable compared to normal vessels. This vascular leakage leads to waste accumulation, increased tumor acidity, and altered metabolism [112]. Moreover, the degree of neovascularization strongly correlates with the potential for distant metastasis. To detect tumor growth, invasion, and metastasis, imaging probes have been developed which target angiogenic factors that are overexpressed on activated ECs but are limited in normal epithelium. These targets include various VEGF isoforms (A–E) [113], VEGFRs, and integrin αvβ3 [114], offering precise tools for visualizing tumor progression.

KDR (also known as VEGFR2), which exhibits a high affinity for VEGF, is significantly upregulated in cancer cells and activated ECs within the TME, driving proangiogenic processes. Suppressing KDR expression has been shown to inhibit tumor-associated angiogenesis. For instance, strong accumulations of ^188^Re-labeled VEGF_189_ peptides were observed in KDR-overexpressing SKOV3 tumors, highlighting its potential for KDR-targeted imaging and therapy [115]. Similarly, VEGF_125–136_, the first identified inhibitor of VEGFA [116], was labeled with copper-64 for PET imaging, showing efficacy in melanoma-, glioblastoma-, and breast cancer-bearing murine models [117]. Furthermore, the humanized anti-VEGFR2 monoclonal antibody Ramucirumab was radiolabeled with copper-64 for PET imaging, demonstrating its specificity in non-small cell lung cancer (NSCLC) mouse models [118].

Further developments include VEGFR1- and VEGFR2-specific imaging probes, ^89^Zr-labeled scVR1/Zr and scVR2/Zr, respectively, which allow selective imaging of VEGFR1^+^ and VEGFR2^+^ cells at different stages of cancer progression [119]. VEGFR expression is tightly associated with the formation of pre-metastasis niches. For instance, VEGFR1^+^CD133^+^ hematopoietic progenitor cells (HPCs) have been implicated in promoting lung metastasis in subcutaneous MDA-MB-435 breast cancer-bearing mice [120]. To image the pre-metastasis niche, an ^18^F-labeled VEGFR1-specific single-chain VEGF mutant, [^18^F]AlF-NODA-scVR1 was developed to target VEGFR1^+^ cells. PET/CT imaging revealed increased lung signals that co-localized with IVIS/CT images. Additionally, autoradiography hot spots demonstrated high VEGFR1 intensity, corroborated by immunofluorescence staining. The study also identified the gradual recruitment of VEGFR1^+^CD133^+^ HPCs to the lungs as metastases developed in the orthotopic 4T1 mouse model [121].

Activated ECs also highly expressed integrin αvβ3, indicating that RGD peptides may be potential probes for neovascular imaging [122]. Lui et al. developed an [^89^Zr]Zr-DFO-heterodimeric peptide, iRGD-PEG_3_-(lys-[^89^Zr]Zr-DFO)-PEG_3_-^D^A7R, which showed specific targeting of integrin αvβ3 and VEGFR2. Both PET imaging and biodistribution data showed the probe could be retained in tumors for over 3 h [123]. Additionally, a [^64^Cu]Cu-DOTA-cRGD(D-BPA)K probe was designed for PET imaging and BNCT application [124]. Based on the success of FAPI probes, a dual-targeting PET probe, [^68^Ga]Ga-FAPI-RGD, was developed. Elevated uptake of [^68^Ga]Ga-FAPI-RGD was detected in both primary tumors and metastases across various cancer types in patients. Compared to ^18^F-FDG and [^68^Ga]Ga-FAPI-46, [^68^Ga]Ga-FAPI-RGD exhibited higher sensitivity and superior diagnostic accuracy, making it a promising tool for cancer detection and monitoring [125].

The processes of vasculogenesis, angiogenesis, and vascular mimicry in the TME play critical roles in sustaining tumor growth, promoting metastasis, and driving treatment resistance. Advances in imaging probes targeting angiogenic factors like VEGF, VEGFRs, and integrins have significantly enhanced the visualization of dynamic changes within the tumor vasculature. These innovations not only improve cancer detection and monitoring of disease progression but also hold therapeutic potential when coupled with appropriate radioisotopes, enabling cancer theranostics.

#### 3.2.2. Lymphatic Vessels

The lymphatic system, consisting of lymphatic vessels and lymphatic organs [126], maintains fluid balance in the body by draining excess tissue fluids and particulate matter from the bloodstream. Additionally, lymphatic vessels also help immune cells migrate to lymphoid tissues, thereby supporting immune surveillance [127,128]. A higher density of lymphatic vessels known as lymphangiogenesis develops around primary tumors, offering alternative routes for tumor dissemination [129]. This process is closely associated with metastasis and poor prognosis in cancer patients [130,131]. Within TME, lymphatic vessels are often dilated, irregular, and leaky, leading to inefficient lymphatic drainage, impaired drug delivery, and increased metastatic potential.

Radiolabeled [^99m^Tc]Tc-sulfur colloid, [^99m^Tc]Tc-phytate, and [^99m^Tc]Tilmanocept (Lymphoseek^®^) are routinely used for lymph node scanning in the clinic. These imaging probes, which can operate on the nanoscale, are effective at entering and being retained within lymphatic vessels and provide high-resolution lymphatic imaging [132,133,134,135]. Thorek et al. developed [^89^Zr]Zr-ferumoxytol, a formulation of iron oxide particles modified with DFO, which enables multimodal PET and MRI of lymph nodes in prostate cancer-bearing mice [136].

Key proteins involved in lymphangiogenesis, such as prospero homeobox 1 (Prox1) [137], podoplanin (PDPN) [138], and lymphatic vessel endothelial hyaluronic acid receptor 1 (LYVE-1) [139], are essential for the formation of lymphatic networks. Prox1 upregulates the expressions of integrin α9 and VEGFR-3 on lymphatic ECs. When activated by its ligands, VEGF-C and VEGF-D, VEGFR3 promotes the maturation of lymphatic vessels [140,141,142]. In the TME, cancer cells express high levels of neuropilin-2, VEGF-A, VEGF-C, and VEGF-D, which stimulate lymphangiogenesis and increase the potential for lymphatic spread [143,144].

Imaging advances have leveraged these lymphatic markers for cancer detection. Kwon et al. injected ICG intradermally to visualize early-stage lymphatic leakage in B16F-10 tumors overexpressing VEGF-C. Their findings suggest that tumor-secreted VEGF-C destabilizes lymphatic vessels, which promotes cell extravasation and lymphatic spread [145]. In addition, another NIRF tracer, P20D680, a poly (ethylene glycol) (PEG)-based lymphatic tracer, has shown potential in evaluating lymphangiogenesis and lymphatic vessel contractility [146,147].

PDPN is essential for the formation of lymphatic vessels and has been found to be elevated in several cancers [126]. Mouse embryos lacking PDPN exhibit a phenotype characterized by the mixing of blood and lymphatic vessels, which results in blood-filled lymphatic vessels and severe edema due to the abnormal separation between blood and lymphatic vessels [148]. Given the significance of PDPN in lymphangiogenesis and tumor development, Kato et al. developed an IR700-conjugated anti-PDPN antibody that is suitable for both fluorescence imaging and near-infrared photoimmunotherapy [149].

LYVE-1, primarily found on lymphatic ECs, is involved in the transport of hyaluronan, a component abundant in the TME [150]. Elevated LYVE-1 expression is associated with lymphangiogenesis and the spread of metastasis to lymph nodes [151,152]. Anti-LYVE-1 immuno-PET, using the [^125^I]I-anti-LYVE-1 antibody, has shown superior performance to the conventional [^18^F]FDG/PET scan in detecting metastasis, as it captures lymphangiogenesis more effectively [153].

Although [^125^I]I-anti-VEGFR-3 antibodies accumulate specifically in lymphatic vessels, their ability to inhibit lymphangiogenesis presents challenges for imagining biological processes without interfering with them [153]. This issue could be addressed by using peptide-based probes that specifically target VEGFR-3, such as [^68^Ga]Ga-DOTA-TMVP1 and [^68^Ga]Ga-DOTA-TMVP1448. Both probes have demonstrated excellent selectivity in vivo by targeting tumor-induced metastatic lymph nodes while sparing contralateral lymph nodes. Moreover, [^68^Ga]Ga-DOTA-TMVP1448 also provides superior imaging resolution compared to [^18^F]FDG [154,155].

Lymphangiogenesis and lymphatic vessel remodeling are crucial processes that influence cancer metastasis and patient prognosis. Imaging the lymphatic system with probes targeting lymphatic markers, such as PDPN, LYVE-1, and VEGFR-3, offers non-invasive methods to assess lymphatic vessel activity and lymph node metastasis, thus offering valuable insights for predicting therapeutic response.

### 3.3. Immunosuppressive Cells

Interactions between immune cells and cancer cells play dual roles in oncogenesis and tumor progression [156]. Within the TME, immune cells can adopt pro-tumorigenic or anti-tumorigenic phenotypes depending on the context [157]. This section highlights recent advances in molecular imaging probes targeting various immune cells in the field of cancer research.

Immune cells within the TME can shift toward a pro-tumorigenic phenotype, fostering a favorable environment for rapid growth and potential immune evasion [157]. Myeloid cells, for instance, are recruited to the TME and may initially mediate anti-tumor inflammation, suppressing tumor growth during early tumorigenesis [158]. However, excessive cytokine release within the TME can induce immature myeloid cells to infiltrate the TME and differentiate into myeloid-derived suppressor cells (MDSCs), which suppress immune responses and promote tumor progression [159]. Hoffmann et al. radiolabeled polymorphonuclear (PMN-) and monocytic (M-) MDSCs with ^64^Cu-NOTA-anti-CD11b monoclonal antibodies to study their dynamics in vivo using PET imaging in breast cancer and melanoma mouse models. PET imaging revealed that both PMC- and M-MDSCs were recruited to primary tumors and lung metastases in both models, with recruitment strongly correlating with tumor progression [160].

Additionally, Zinnhardt et al. developed an [^18^F]F-DPA-714 TSPO probe for glioma imaging. This probe revealed the high TSPO expression in HLA-DR^+^ glioma-associated myeloid cells, including CD45^med^CD14^+++^ MDSCs and CD45^high^CD14^++^ TAMs. Moreover, more intense DPA-714 signals were detected in grade IV glioblastoma lesions compared to grade II oligodendroglioma, indicating the potential of this probe in staging gliomas [161]. S100A8/A9, an exosomal protein released by MDSCs, interacts with pattern recognition receptors RAGE and TLR4, initiating an autocrine loop that promotes MDSC recruitment in pre-metastatic sites. SPECT/CT imaging demonstrated elevated uptake of ^111^In-DTPA-αS100A9 in the lungs, spleen, and tumors of mice implanted with the lung-metastatic 4T1.2 breast cancer cells, compared to non-metastatic 67NR cells [162]. The spleen uptake of [^111^In]In-DTPA-αS100A9 correlated well with elevated Gr-1^+^CD115^+^CCR2^high^CX3CR1^low^ monocytes, which release S100A8/A9 in response to CCL2 secreted by metastatic cancer cells. Interestingly, an increased [^111^In]In-DTPA-αS100A9 signal was spotted in the lungs 10 days after tumor implantation, whereas tumor signals in the lungs were only detectable on day 20. This finding underscores the potential of ^111^In-DTPA-αS100A9 as a potential imaging probe for detecting pre-metastatic niches.

MDSCs play a pivotal role in immune evasion by suppressing T cell and NK cell functions [163] and promoting the differentiation of regulatory T cells (Tregs) through the TGF-β and IL-10 pathways [164]. Tregs, a specialized subset of CD4^+^ T cells, are essential for maintaining self-tolerance and regulating immune responses [165]. Compared to natural Tregs (nTregs) originating from the thymus, induced Tregs (iTregs) are differentiated in the TME in response to high levels of TGF-β and IL-10 [166,167].

Early imaging strategies for Tregs included the transduction of murine Tregs with the human sodium/iodide symporter (NIS) and labeling with ^99m^TcO4^−^ for in vivo visualization [168]. Jacob et al. differentiated Tregs from peripheral blood mononuclear cells derived from healthy volunteers and then labeled them with [^89^Zr]Zr-oxine. Their findings demonstrated a 4-fold higher uptake of [^89^Zr]Zr-oxine in Tregs cultured in Hank’s Balanced Salt Solution compared to standard X-Vivo15 medium [169]. Additionally, [^89^Zr]Zr-Df-Bz-NCS-anti-CD3 mAb has been used to visualize human T cell expansion in a graft-versus-host disease mouse model receiving human peripheral blood mononuclear cell transfusion. Notably, the signal correlated with T cell numbers and decreased in groups receiving syngeneic Treg transfers [170]. While these imaging probes have been validated in transplantation models, their application in tumor-bearing models remains underexplored, leaving a gap in Treg-targeted imaging in oncology.

Macrophages, differentiated from monocytes, exhibit remarkable plasticity and can be polarized into classically activated M1 macrophages (tumor killing and defense roles) or alternatively activated M2 macrophages (wound healing, tissue remodeling, and tumor progression) based on the cytokine milieu [171]. Tumor-associated macrophages (TAMs), predominantly classified under the M2 phenotype, represent the primary macrophage population in many cancers and are pivotal in tumor progression [172]. TAMs promote angiogenesis by secreting VEGF [173], induce Treg differentiation through IL-10 and TGF-β secretion [171], and are recruited to tumor sites by tumor-derived CSF-1 [174]. Their activity often compromises the efficacy of cancer therapies. Consequently, imaging TAMs within the TME could significantly aid in the development of new therapeutics.

The macrophage mannose receptor (MMR, or CD206) is a key M2 marker and is primarily expressed on MHC II^low^ CD11b^+^ cells, especially TAMs. CD206 is a reliable target for in vivo identification of M2-like TAMs [175]. MMR-positive macrophages are immunosuppressive and angiogenic, contributing to tumor progression and correlating with poor prognosis in cancer patients [171,176]. To leverage CD206 as a molecular imaging target, an MMR-specific nanobody, [^99m^Tc]Tc-αMMR Nb, was developed using an immunized alpaca. This probe demonstrated high specificity for MHC II^low^ TAMs in wild-type 3LL-R lung carcinoma-bearing mice. Notably, tumor uptake of [^99m^Tc]Tc-αMMR Nb was significantly reduced in CCR2-deficient mice, which exhibit impaired TAM infiltration, confirming the specificity of the probe and dependence on TAM presence [175]. However, high liver and spleen accumulation of [^99m^Tc]Tc-αMMR Nb prompted the development of a bivalent αMMR-αMMR Nb, which is preferentially blocked in the liver and spleen. Administering a 20-fold excess of bivalent Nbs mixed with [^99m^Tc]Tc-αMMR Nbs effectively reduced liver and spleen uptake, improving the tumor-to-tissue ratio.

To advance TAM-targeting imaging tools for clinical translation, Blykers et al. developed camelid single-domain antibody fragments (sdAbs) with dual cross-reactivity to mouse and human MMR. The anti-MMR 3.49 sdAb was radiolabeled to create both [^99m^Tc]Tc-αMMR 3.49 and [^18^F]FB-αMMR 3.49 tracers, which were evaluated in wild-type C57BL/6, MMR-deficient, and CCR2-deficient mice inoculated with 3LL-R Lewis lung carcinoma cells [177]. Both nanobodies demonstrated comparable tumor-targeting efficiency, achieving approximately 2.4% injected radioactivity (IA)/g in tumor tissue. However, [^99m^Tc]Tc-αMMR 3.49 exhibited 20-fold higher kidney accumulation compared to [^18^F]FB-αMMR 3.49 at the 3 h time point in 3LL-R lung carcinoma-bearing mice. This increased kidney accumulation is attributed to the lysosomal trapping of radiometal-labeled compounds in renal cells, a limitation not observed with fluorinated tracers [178]. Moreover, PET imaging with fluorine-18 offers superior sensitivity and spatial resolution compared to SPECT imaging with technetium-99m, positioning [^18^F]FB-αMMR 3.49 as a more suitable candidate for clinical applications. These findings highlight the potential of fluorine-based nanobody tracers for enhanced tumor imaging and improved clinical utility.

CD163 is another marker selectively found on the surface of the M2-like macrophages. A peptide-based probe, [^18^F]AlF-NODA-MP-C6-CTHRSSVVC, successfully targeted CD163-positive TAMs in cyclophosphamide-treated CT26 tumor-bearing mice, demonstrating its specificity [179]. Furthermore, the [^64^Cu]Cu-ICT-01 probe, initially developed for atherosclerosis imaging, exhibited high specificity for CD163 and holds potential for future applications in cancer imaging [180].

Inspired by the interaction of macrophages with high-density lipoprotein (HDL), two ^89^Zr-labeled reconstituted HDL (rHDL) nanotracers—[^89^Zr]Zr-apoA-I-labeled rHDL ([^89^Zr]Zr-AL-rHDL) and [^89^Zr]Zr-phospholipid-labeled rHDL ([^89^Zr]Zr-PL-rHDL)—were designed to image TAMs in orthotopic 4T1 breast cancer mouse models. Both tracers demonstrated similar tumor accumulation at 48 h and co-localization with Iba-1 signals at 24 h, as confirmed by ex vivo autoradiography [181]. Despite differing biodistribution profiles—[^89^Zr]Zr-AL-rHDL had prolonged blood circulation and higher kidney uptake, while [^89^Zr]Zr-PL-rHDL showed greater bone retention—flow cytometry analyses revealed that both tracers were preferentially taken up by TAMs over other immune cells such as dendritic cells, T cells, and monocytes. This underscores their potential utility in clinical TAM imaging.

Immunosuppressive cells within the TME, including MDSCs, Tregs, and TAMs, play pivotal roles in promoting immune evasion. Advances in molecular imaging probes targeting these cells have facilitated non-invasive visualization of immune cell dynamics within the TME. Probes for MDSC markers, Treg-specific molecules, and TAM markers such as CD206 and CD163 have shown encouraging results in preclinical studies. These imaging tools enhance our understanding of tumor–immune interactions and offer methods to monitor therapeutic responses. The simultaneous use of multiple probes to target diverse immune cell populations could offer a comprehensive view of the immunosuppressive landscape, paving the way for improved cancer diagnostics and precision immunotherapy.

## 4. Conclusions

The tumor microenvironment (TME) represents a complex and dynamic ecosystem that plays a crucial role in cancer progression, metastasis, and resistance to therapy. Comprised of cancer cells and diverse stromal components—including the extracellular matrix (ECM), cancer-associated fibroblasts (CAFs), vascular and lymphatic networks, and immunosuppressive immune cells—the TME orchestrates a supportive niche for tumor development.

**Figure 2:**
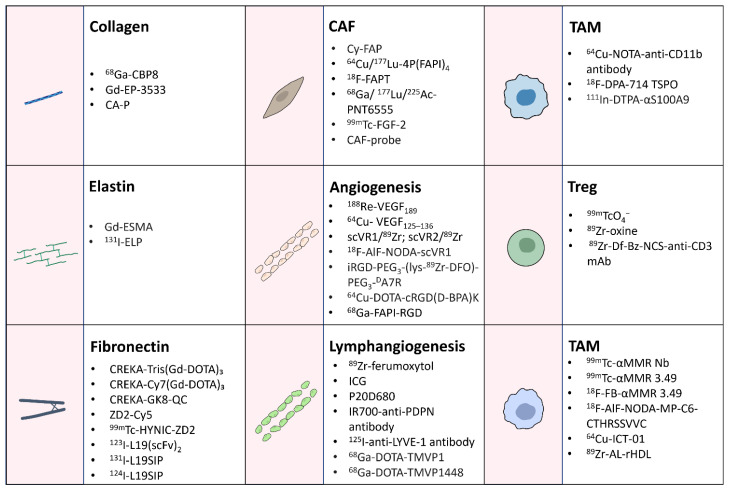
Imaging probes targeting various components of the tumor microenvironment. These imaging tools enable a comprehensive understanding of the TME, supporting diagnostic and therapeutic advancements by targeting ECM, stromal cells, vascular and lymphatic networks, and immune cell populations. Probes for ECM visualization focus on collagen, elastin, and fibronectin. CAF probes target markers such as FAP and FGF-2 to assess stromal cell activity. Vascular and lymphatic network imaging utilizes probes targeting angiogenesis and lymphangiogenesis markers, including VEGF, VEGFRs, podoplanin, and LYVE-1. Immune-targeted probes, specific to MDSCs, TAMs, and Tregs, provide insights into the immunosuppressive cell dynamics within the TME.

Recent advancements in molecular imaging of the TME have greatly enhanced our understanding of tumor biology (Figure 2). These imaging techniques allow for non-invasive assessments of ECM remodeling, stromal cell activity, vascular and lymphatic dynamics, and interactions among immune cells. In addition to imaging, radiopharmaceutical therapies using isotopes such as Lutetium-177, copper-67, lead-212, bismuth-212, actinium-225 are emerging as promising strategies to target the TME and cancer cells with high specificity to eradicate cancer cells and prevent metastasis potentially. These therapies specifically deliver radiation doses to tumors while minimizing off-target effects [182,183,184]. By combining imaging and therapeutic capabilities, these isotopes enable theranostic approaches that integrate diagnosis, treatment, and monitoring into a single platform.

However, the clinical translation of novel imaging probes faces several regulatory challenges. These include stringent requirements for demonstrating safety, efficacy, and reproducibility in human studies, as mandated by regulatory agencies such as the FDA and EMA. The high costs and lengthy timelines of preclinical and clinical trials complicate drug development. A key challenge is the need for standardized probe production and quality control, especially for probes targeting the complex and often poorly understood components of the tumor TME. Additionally, integrating these probes into clinical workflows may require updated imaging hardware, software, and personnel training, posing logistical challenges for widespread adoption.

Despite these hurdles, integrating artificial intelligence (AI) may further increase detection specificity and sensitivity, reducing the time for imaging processing and filing reports, and increasing the throughput. Additionally, more information can be extracted from the obtained images using radiomics, a technique that quantifies imaging features to reveal patterns not visible to the human eye. This method correlates features with multi-omics data.

Radiomics provides a non-invasive way to assess tumor heterogeneity, offering biomarkers for disease characterization, prognosis prediction, and treatment response evaluation. When combined with advanced molecular imaging, these technologies can provide unprecedented insights into tumor progression and response to therapy. The continuous refinement and clinical application of imaging probes can transform cancer management by enhancing diagnostic accuracy and allowing for better monitoring of treatment responses. Additionally, AI techniques provide priceless support in expediting regulatory approval by improving the interpretability of imaging data and aiding in validating robust biomarkers.

Future efforts should address regulatory, technical, and accessibility barriers through interdisciplinary collaboration and public–private partnerships. Clearer regulatory and approval processes, along with global standardization, are essential for fully realizing the clinical potential of novel imaging probes. These developments enhance diagnostic accuracy and allow for more effective monitoring of treatment responses, ultimately leading to improved patient outcomes.

## Figures and Tables

**Table 1:** Imaging techniques mentioned in the present article.

Imaging Modality	Advantages	Limitations
Positron Emission Tomography (PET)	▪ High sensitivity ▪ Quantifiable molecular data ▪ Real-time functional imaging	▪ Lack of anatomical information ▪ Radiation exposure ▪ High cost of radiotracers
Single Photon Emission Computed Tomography (SPECT)	▪ High sensitivity ▪ Wider availability ▪ Relatively cost-effective compared to PET	▪ Lack of anatomical information ▪ Radiation exposure ▪ Lower resolution than PET
Magnetic Resonance Imaging (MRI)	▪ Non-ionizing radiation ▪ High soft-tissue contrast ▪ Functional and molecular information	▪ High cost ▪ Long acquisition time ▪ Not suitable for patients who have metal-based implants
Computed Tomography (CT)	▪ Excellent anatomical information ▪ Short imaging acquisition time ▪ Widely available	▪ Radiation exposure ▪ Poor soft-tissue contrast ▪ Potential nephrotoxicity may occur due to the use of contrast agents.
Ultrasound	▪ Non-radiation ▪ Real-time imaging ▪ Portable and cost-effective	▪ Operator dependency ▪ Limited penetration depth ▪ Poor resolution and scatter due to the presence of air in tissues
Fluorescence Imaging	▪ Non-radiation ▪ High molecule specificity when combined with targeted probes ▪ Real-time surgical guidance	▪ Insufficient tissue penetration ▪ High autofluorescence background within visible and Near-Infrared-I (NIR-I) window

## Data Availability

Not applicable.
